# Minimum Detection Concentration of Hydrogen in Air Depending on Substrate Type and Design of the 3ω Sensor

**DOI:** 10.3390/s23219009

**Published:** 2023-11-06

**Authors:** Dong-Wook Oh, Kwangu Kang, Jung-Hee Lee

**Affiliations:** 1 Department of Mechanical Engineering, Chosun University, Gwangju 61452, Republic of Korea; 2 Offshore Industries R&BD Center, Korea Research Institute of Ships & Ocean Engineering, Geoje 53201, Republic of Korea; kgkang@kriso.re.kr (K.K.); jhlee@kriso.re.kr (J.-H.L.)

**Keywords:** 3 omega method, gas thermal conductivity, hydrogen concentration, minimum detection concentration

## Abstract

Hydrogen has emerged as a promising carbon-neutral fuel source, spurring research and development efforts to facilitate its widespread adoption. However, the safe handling of hydrogen requires precise leak detection sensors due to its low activation energy and explosive potential. Various detection methods exist, with thermal conductivity measurement being a prominent technique for quantifying hydrogen concentrations. However, challenges remain in achieving high measurement sensitivity at low hydrogen concentrations below 1% for thermal-conductivity-based hydrogen sensors. Recent research explores the 3ω method’s application for measuring hydrogen concentrations in ambient air, offering high spatial and temporal resolutions. This study aims to enhance hydrogen leak detection sensitivity using the 3ω method by conducting thermal analyses on sensor design variables. Factors including substrate material, type, and sensor geometry significantly impact the measurement sensitivity. Comparative evaluations consider the minimum detectable hydrogen concentration while accounting for the uncertainty of the 3ω signal. The proposed suspended-type 3ω sensor is capable of detecting hydrogen leaks in ambient air and provides real-time measurements that are ideal for monitoring hydrogen diffusion. This research serves to bridge the gap between precision and real-time monitoring of hydrogen leak detection, promising significant advancements in the related safety applications.

## 1. Introduction

International climate agreements have spurred active research and development in renewable and clean energy sources, primarily motivated by the need to curb fossil fuel usage and mitigate greenhouse gas emissions. Hydrogen, an environmentally friendly fuel, has garnered substantial attention for its capacity to replace fossil fuels and emit only water vapor upon combustion, rendering it a notable carbon-neutral alternative. Hydrogen serves as both a heat source, replacing conventional fossil fuels, and a direct electricity generating source through fuel-cell technology. It is imperative to advance technologies for liquefaction and storage of substantial hydrogen quantities to facilitate the widespread adoption of hydrogen [1,2,3]. However, handling hydrogen requires vigilance, as even minor leaks into the atmosphere can lead to explosions due to hydrogen’s low activation energy. Hence, developing precise hydrogen leak detection sensors takes precedence in establishing safety measures for hydrogen usage [4,5,6,7].

Various methodologies for detecting hydrogen leakage into the atmosphere have been proposed. These methodologies leverage a spectrum of measurement principles with unique advantages and limitations contingent upon specific operational conditions. The pinnacle of precision in hydrogen leak detection is achieved through gas chromatography or mass spectrometry [6]. However, it is essential to note that these sophisticated instruments primarily find their niche within controlled laboratory settings, rendering them less amenable to real-time and on-site measurement. In practice, the prevailing techniques for hydrogen sensors pivot on the principles of electrochemistry and catalysis [6,7,8,9,10]. These methodologies demonstrate remarkable sensitivity within the low hydrogen concentration range, spanning from hundreds to thousands of parts per million (ppm). Nevertheless, it is imperative to recognize that these techniques tend to exhibit signal saturation as hydrogen concentrations reach few percent. Consequently, supplementary sensor methodologies are often concurrently employed for precisely quantifying hydrogen concentrations over 1% [6].

Thermal conductivity measurement is a widely adopted technique for quantifying hydrogen concentrations in the field of research and experiments. Hydrogen’s thermal conductivity exceeds that of air by seven times at standard room temperature. This distinctive attribute allows for determining hydrogen concentrations by measuring the thermal conductivity of a mixture of air and hydrogen [11]. The thermal conductivity sensor has a comparatively lower degree of operational constraints when compared to electrochemical or catalytic-type sensors, which necessitate the presence of oxygen for their functionality. The advantages of the thermal conductivity sensors encompass swift response time and minimal power consumption. It also exhibits enhanced stability and reproducibility due to its inherent simplicity, coupled with the sensor’s resistance to contamination, a prevalent limitation in other sensor technologies [12].

Nevertheless, challenges in selectivity may arise when dealing with gases such as He, CH_4_, CO, characterized by higher thermal conductivities than air. Thermal conductivity sensors show diminished measurement sensitivity with moisture and humidity within the sample gas at elevated temperatures [13]. Additionally, temperature correction may be necessary, and the measurement accuracy tends to decrease at low hydrogen concentrations below 1%. Despite these considerations, the thermal conductivity sensors remain the preeminent choice for precise hydrogen concentration measurements within experimental and engineering systems [6].

Electrochemical or catalytic sensors are typically the primary choice for hydrogen leak detection. While effective at signaling the presence of hydrogen, these sensors have limitations in assessing criticality in the field, especially when hydrogen levels are near the lower explosive limit of 4%. Moreover, they cannot confirm whether hydrogen concentrations are consistently below this threshold, making it challenging to take precise corrective actions. Consequently, there is an urgent need to develop thermal conductivity sensors with enhanced sensitivity and superior spatial and temporal resolution, particularly for detecting hydrogen concentrations in the sub-percent range. Achieving this goal necessitates sensitivity enhancement designs to lower the minimum measurable hydrogen concentration to below 1%.

Recent research has explored the application of the 3ω method for measuring hydrogen concentrations in ambient air [11,12,13,14]. The 3ω method, widely used for thermal conductivity measurements, extends its versatility beyond thin films and substrates to encompass liquids and gases [15,16]. Utilizing 3ω sensors opens the door to sub-micron spatial resolution and time constant below a few ms. Furthermore, it can eliminate the need for conventional processes such as sample drying and pre-conditioning, which typically involve temperature adjustments and are common in conventional hydrogen sensors. 

In this research, we performed a thermal analysis on the sensor design variables to reduce the minimum detectable hydrogen concentration in ambient air using the 3ω method. We employed theoretical calculations to determine measurement signals influenced by factors such as substrate material, type, and sensor geometry, which significantly impact thermal conductivity measurement sensitivity. Additionally, we conducted a comparative evaluation, considering the measurable minimum hydrogen concentration while considering uncertainty errors affecting the 3ω signal. The 3ω sensor proposed in this study can detect hydrogen leaks in ambient air and provide real-time measurements of hydrogen concentrations suitable for monitoring of hydrogen diffusion. This advancement holds great promise for enhancing safety measures and optimizing hydrogen-related applications.

## 2. Thermal Modeling of 3ω Sensors

**Figure 1:**
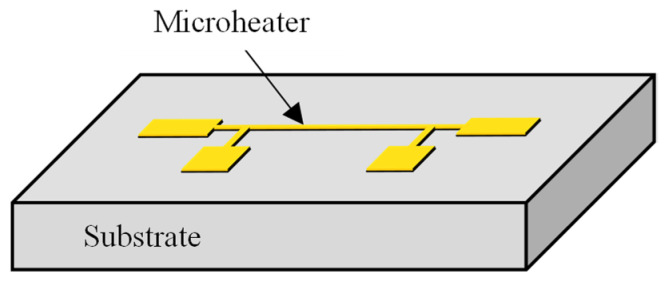
Schematic of a 3ω sensor with a microheater on a substrate.

The 3ω method measures the temperature amplitude and phase lag in response to alternating current (AC) applied to a microheater. This method enables the determination of the thermal properties of the surrounding medium by fitting the measured data with the theoretical equation. A metal microheater is deposited and patterned onto a substrate in a typical 3ω sensor configuration, as illustrated in Figure 1. When an AC is applied to this microheater, the temperature oscillates following a sine wave pattern. By comparing the amplitude of the temperature oscillation and the phase lag relative to the original AC signal with theoretical equations, it becomes possible to calculate thermal properties such as the thermal conductivity or thermal diffusivity of the substrate. The theoretical expression governing the temperature oscillation of the microheater is as presented in the equation below [17]:

(1) $$∆T=\frac{\dot{Q}}{\pi l}\int_{0}^{\infty }\frac{1}{\gamma }\times \frac{{sin}^{2}\left(xb\right)}{{\left(xb\right)}^{2}}dx$$

Here, Δ*T* represents the temperature oscillations of the microheater, and $\dot{Q}$ is the generated heat flux. *l*, 2*b*, ω, and *x* correspond to the microheater’s length, width, angular frequency, and integration variable, respectively. *γ* can be expressed as $k\sqrt{x^{2}+i\frac{2\omega }{\alpha }}$, where *k* and *α* denote the thermal conductivity and thermal diffusivity of the substrate, respectively. The temperature oscillations of the microheater are in the form of a complex number with amplitude and argument representing temperature amplitude and phase lag, respectively [18]. One of the most significant advantages of the 3ω method is its ability to achieve very high spatial resolution by adjusting the thermal penetration depth (TPD) based on the AC frequency. TPD refers to the physical distance over which temperature oscillations generated by the heater are transmitted into the substrate. It can be expressed as $\sqrt{\left(\alpha ⁄2\omega \right)}$. For instance, in the case of a SiO_2_ substrate, at AC frequencies ranging from a few Hz to kHz, TPD can vary from several hundred nanometers to a hundred micrometers. Thus, the thickness of the substrate to be measured is determined based on the frequency range, allowing for precise spatial resolution control. This capability facilitates thermal analysis of ultra-thin films or localized regions within samples [19,20].

**Figure 2:**
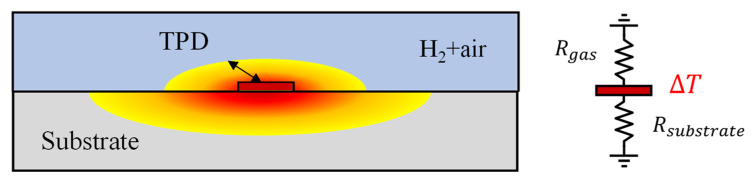
Schematic of a 3ω sensor and a thermal resistance circuit of the substrate and gas mixture.

When dealing with fluid samples, conducting measurements using the bi-directional 3ω method is feasible. This approach employs a metal microheater, deposited on a substrate with well-known thermal properties [15,21]. However, it is imperative to operate assuming that the measured fluid remains static, without any convective heat transfer effects, such as natural convection. Figure 2 illustrates this configuration, with the microheater sandwiched between the sample gas and the sensor substrate. The temperature oscillations can be expressed as in the following equation [20].

(2) $$∆T=\frac{\dot{Q}}{\pi l}\int_{0}^{\infty }\frac{1}{\gamma +\gamma_{gas}}\times \frac{{sin}^{2}(xb)}{{\left(xb\right)}^{2}}dx$$

The subscript “*gas*” denotes the sample gas layer’s properties. With the equation mentioned above, applying the 3ω method has become feasible for the thermal analysis of various gases, where microheater deposition through semiconductor processes was impossible. As mentioned earlier, for air, the TPD is typically less than a few tens of micrometers, contrasting with the SiO_2_ substrate. Consequently, it is possible to accurately measure the thermal conductivity and hydrogen concentration in gas mixtures, such as air, using the 3ω method with just samples with thicknesses exceeding ~0.1 mm [18].

Several crucial considerations come into play when utilizing the bi-directional 3ω method for gas measurement. The thermal conductivity of gases, excluding cases like hydrogen and helium, generally falls below ~0.1 W·m^−1^·K^−1^, one or more orders of magnitude lower than that of typical 3ω sensor substrates like SiO_2_. Selecting a substrate with higher thermal resistance is essential to enhance sensitivity in measuring such low thermal conductivity samples. For example, we consider a 3ω sensor on a SiO_2_ substrate for measuring the thermal conductivity of a gas with 1/10 of the thermal conductivity compared to the substrate. The heat generated by the microheater flows through the substrate and the gas in a parallel thermal resistance circuit, as shown on the right side of Figure 2. Because the thermal resistance of the substrate is one-tenth that of the gas side, most of the heat generated in the microheater flows to the substrate. Consequently, the sensitivity to the thermal conductivity of the gas, and thus the measurement accuracy for changes in hydrogen concentration, is diminished [11]. 

To enhance the measurement sensitivity and accuracy of a gas sample, the thermal resistance of the substrate must be increased to a level comparable to that of the gas sample. One approach is to use materials such as polymers (e.g., polyimide) with thermal conductivities approximately one-fifth of that of SiO_2_ as the substrate of the 3ω sensor. Alternatively, for an even more significant enhancement, etching can remove the substrate entirely, leading to a suspended microheater structure. In the case of a suspended sensor, heat generated by the microheater flows equally to both the upper and lower sides occupied by gas, necessitating the addition of a factor of 2 in the denominator of Equation (1). The configuration of a suspended sensor offers the most sensitive and accurate measurement of the thermal conductivity of gas and is expected to provide the lowest detectable hydrogen concentrations.

In this study, the design parameters of the 3ω sensor were analyzed to assess the minimum detectable hydrogen concentration in the air. Various configurations of 3ω sensors were considered, including SiO_2_ and polyimide substrates and suspended-type sensors. SiO_2_ and polyimide substrates were assumed to have a thickness of 500 μm. Additionally, to compare the measurement sensitivity according to the width of the microheater, calculations were conducted for microheater widths of 4, 10, and 40 μm. Among the design parameters of the 3ω sensor, the sensor width is known to have the most significant impact on measurement sensitivity, excluding the substrate type. [11] Hydrogen concentration in air ranged from 0% to 20% mole fraction (mf) for the sample gas. AC frequencies for the microheater were calculated from 0.1 to 100 Hz. Table 1 summarizes the calculation parameters.

**Figure 3:**
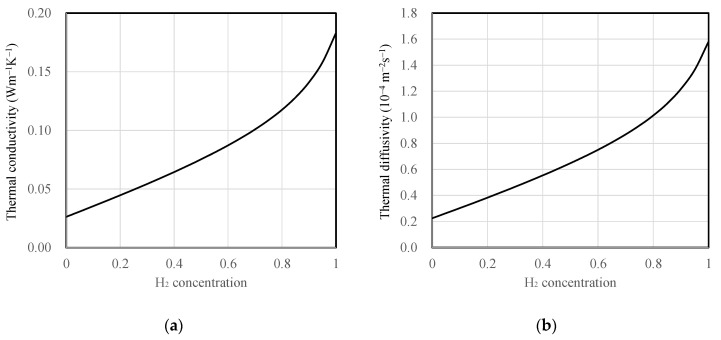
Properties of hydrogen and air mixture depending on hydrogen concentration (mf), (**a**) thermal conductivity and (**b**) thermal diffusivity.

All substrate and mixture gas properties were assumed to be constant regardless of temperature variation. The thermal conductivity of the mixture gas as a function of hydrogen concentration was calculated using the thermal conductivity correlation equation proposed by Mathur for binary gas mixtures [22]. Furthermore, air and hydrogen were treated as ideal gases, and their densities and specific heat capacities were determined as weighted averages based on mole fraction and mass fraction, respectively. The densities were calculated by multiplying the volume fractions with the individual densities of air, 1.16 kg·m^−3^, and hydrogen, 0.0808 kg·m^−3^. The specific heat capacity of dry air at room temperature was 1.007 kJ·kg^−1^·K^−1^ and hydrogen’s specific heat capacity was 14.31 kJ·kg^−1^·K^−1^. These values were averaged by their respective mass fractions to obtain specific heat values as a function of concentration. The thermal conductivity and thermal diffusivity of the mixture gas as a function of hydrogen concentration are depicted in Figure 3. For the substrate materials, SiO_2_ and polyimide were assumed to have thermal conductivities of 1.2 and 0.17 W·m^−1^·K^−1^, and thermal diffusivities of 7.2 × 10^−7^ and 1.1 × 10^−7^ m^2^·s^−1^, respectively.

## 3. Calculation Results

**Figure 4:**
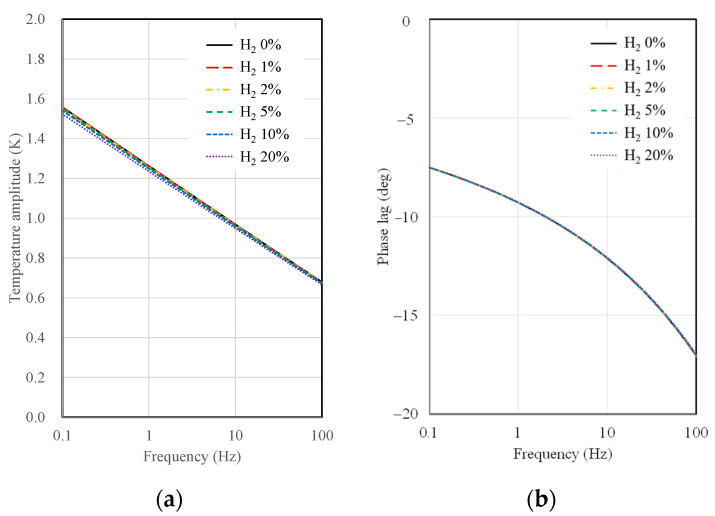
Calculation results of (**a**) temperature amplitude and (**b**) phase lag depending on AC frequency and hydrogen concentration for 10 μm width 3ω sensor on a SiO_2_ substrate.

This section presents the results of temperature amplitude calculations for 3ω sensors, considering the substrate material and type, AC frequency, the width of the microheater, and hydrogen concentration. Figure 4 shows the results of temperature amplitude and phase lag calculations as a function of AC frequency and hydrogen concentration when a 10 µm wide microheater on a SiO_2_ substrate is used. As observed in Figure 4, the sensitivity of the SiO_2_ substrate sensor to changes in hydrogen concentration is notably low. There is some distinction in temperature amplitude concerning hydrogen concentration, particularly at low frequencies, near 0.1 Hz. However, there is a single curve in the case of phase lag, indicating no discernible difference in the calculated values with varying hydrogen concentrations. As mentioned in the thermal resistance analysis, this behavior suggests that the thermal resistance difference between the SiO_2_ substrate and the gas mixture is significant, resulting in a lack of difference in signals concerning hydrogen concentration. 

To determine the minimum detectable concentration of hydrogen, we focused on analyzing the temperature amplitude signal rather than the phase lag. It is widely known that the typical measurement uncertainty for temperature amplitude in the 3ω measurements is around 2% [23]. Assuming a measurement uncertainty of 2% for the temperature amplitude, we established a criterion for detecting the minimum hydrogen concentration. Specifically, we considered hydrogen concentrations where the temperature amplitude (Δ*T*) deviates by more than 2% from the amplitude calculated for pristine air (Δ*T*_0%_). In other words, we calculated the hydrogen concentration at which the temperature amplitude ratio, Δ*T*/Δ*T*_0%_, averages less than 0.98 across the frequency range of 0.1 to 100 Hz. We deemed this concentration to be the minimum detectable hydrogen concentration using the 3ω sensor. 

**Figure 5:**
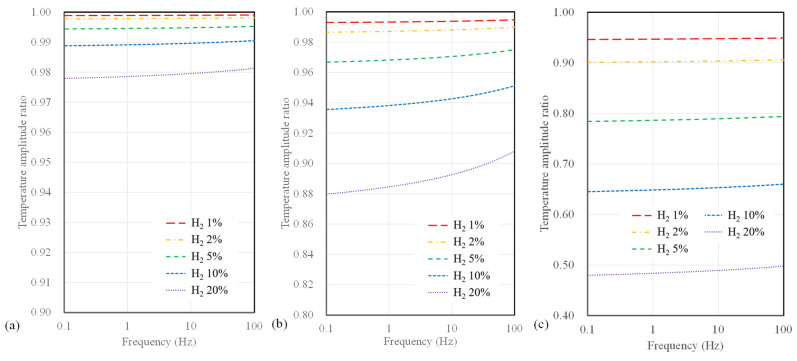
Calculation results of temperature amplitude ratio depending on AC frequency and hydrogen concentration for (**a**) SiO_2_ substrate, (**b**) polyimide substrate, and (**c**) suspended-type sensors.

Next, we present the calculated temperature amplitude ratios for different substrate types. Figure 5 illustrates the temperature amplitude ratios for sensors with a 10 µm width, considering SiO_2_, polyimide substrates, and suspended-type sensors. In all calculations, significant variations in temperature amplitude concerning hydrogen concentration are observed at low frequencies below 1 Hz. Furthermore, it is noteworthy that for SiO_2_ sensors, which exhibit the lowest measurement sensitivity among all substrates, the temperature amplitude only deviates by 2.3% or less, even up to a maximum hydrogen concentration of 20%. In contrast, polyimide substrates and suspended sensors show differences of 12% and 48%, respectively. Among these, the suspended-type sensor displays the highest measurement sensitivity regarding hydrogen concentration, followed by the polyimide and SiO_2_ substrate sensor.

**Figure 6:**
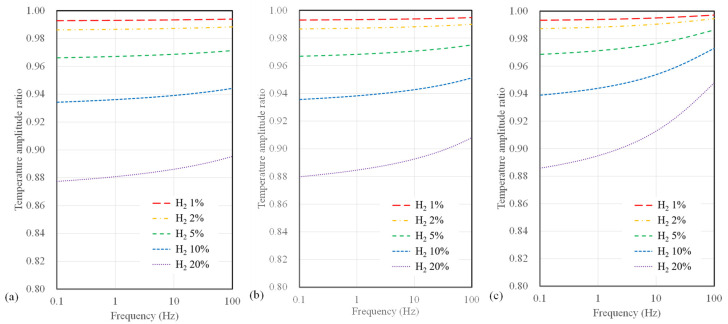
Calculation results of temperature amplitude ratio depending on AC frequency and hydrogen concentration for polyimide substrate sensor with widths of (**a**) 4 μm, (**b**) 10 μm, and (**c**) 40 μm.

Figure 6 illustrates the calculated results of the temperature amplitude ratios concerning hydrogen concentration and AC frequency when varying the microheater width for the 3ω sensor based on a polyimide substrate. The width of the microheater ranges from 4, 10, and 40 µm. In terms of sensor width, it is apparent that the temperature amplitude ratio does not vary significantly compared to the substrate type. However, as the sensor width decreases, an observable increase in sensitivity to hydrogen concentration occurs. Additionally, it is noteworthy that when the sensor width is 4 µm, the change in temperature amplitude ratio concerning frequency was relatively small compared to that observed with larger widths.

**Figure 7:**
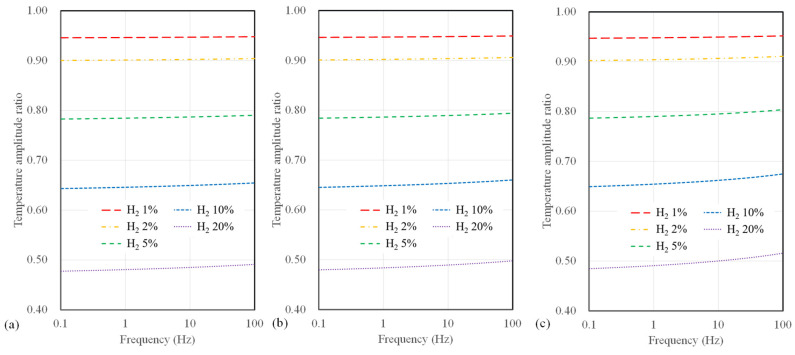
Calculation results of temperature amplitude ratio depending on AC frequency and hydrogen concentration for suspended sensor with widths of (**a**) 4 μm, (**b**) 10 μm, and (**c**) 40 μm.

Conversely, as the width increases, a significant drop in sensitivity at higher frequencies (above 10 Hz) becomes evident. Figure 7 presents the calculated results of temperature amplitude ratios concerning the microheater width for suspended sensors. The sensor width has a minor impact on hydrogen measurement sensitivity for the suspended-type sensor. Notably, a sensor width of 4 µm shows a slight improvement in sensitivity to hydrogen concentration compared to broader width configurations for all substrate types. Furthermore, compared to polyimide substrate sensors, there is a relatively small change in sensitivity to frequency for a suspended sensor.

A standard photolithography technique can achieve a metal microheater with a 4 µm width via the semiconductor process. However, for sensor widths exceeding ~40 µm, an alternative approach, such as employing a shadow mask during the metal deposition process to simultaneously pattern the metal, can significantly simplify the sensor fabrication process. In light of this, when creating a 3ω sensor on a polyimide substrate, it is advisable to fabricate it with the conventional photolithography process whenever possible. This approach allows for a narrower microheater patterning, maximizing the measurement sensitivity. On the other hand, in the case of suspended sensors, where the difference in hydrogen measurement sensitivity based on sensor width is negligible, fabrication using a shadow mask should provide sufficient sensitivity. This choice streamlines production while still meeting the necessary sensitivity requirements.

## 4. Minimum Detectable Hydrogen Concentration

**Figure 8:**
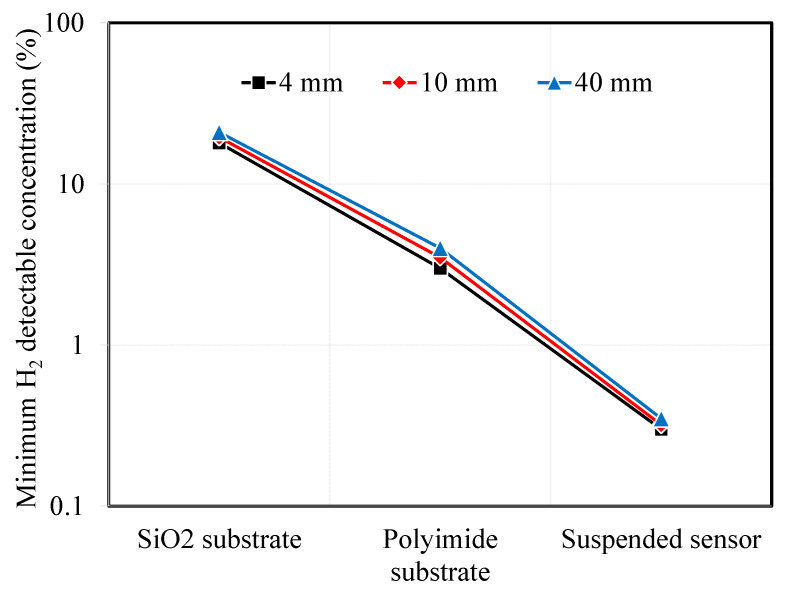
Minimum detectable hydrogen concentration depending on substrate types and sensor width.

In the previous section, we calculated the temperature amplitude and temperature amplitude ratio for 3ω sensors under varying conditions. We considered factors such as the substrate material, type, microheater width, and AC input frequency. It was observed that as the thermal resistance of the substrate increased, the minimum detectable hydrogen concentration decreased. Although the effect was smaller than that of substrate thermal resistance, narrower microheater widths were associated with improved hydrogen measurement sensitivity. Assuming a measurement uncertainty of 2% for the temperature amplitude ratio, we can use the previous calculations to determine the minimum detectable hydrogen concentration. Figure 8 illustrates the calculated minimum hydrogen concentrations in the air that can be detected for the different substrate types and microheater widths of the 3ω sensor.

For the SiO_2_ substrate, the minimum detectable hydrogen concentration was above 18%. To detect a difference in signal compared to pristine air without hydrogen, the thermal conductivity of the mixed gas of air and hydrogen should be at least 0.043 W·m^−1^·K^−1^. Designing microheaters with smaller widths is advantageous, but it was challenging to observe significant differences in the minimum hydrogen detection concentrations between 4 μm and 40 μm sensors, which were 18% and 21%, respectively. On the other hand, when using a polyimide substrate sensor, it is expected that researchers are able to detect the minimum hydrogen concentration, which is the explosion limit, at around 3–4%. Finally, the suspended sensor, which has the lowest minimum detectable hydrogen concentration, is expected to measure hydrogen concentrations from 0.3% and 0.35%.

Two additional considerations can be made apart from substrate type and microheater width to reduce the minimum detectable hydrogen concentration further. As observed in all previous results, the sensitivity to hydrogen concentration measurements was higher at low AC frequencies. Therefore, setting the fitting range of temperature amplitude measurements to 10 Hz or less could increase the sensitivity to hydrogen concentration measurements and lower the minimum detectable concentration. Furthermore, it is possible to reduce the uncertainty of temperature amplitude measurements by taking longer time averages at low frequencies in the 3ω sensor and applying low-frequency noise reduction techniques. Suppose these measures are assumed to reduce the temperature amplitude measurement uncertainty to the 1% level. In that case, the minimum hydrogen detection concentrations for SiO_2_, polyimide, and suspended sensors with a 4 μm microheater width will decrease to 9.2%, 1.4%, and 0.14%, respectively. In other words, in the case of suspended-type sensors, the minimum detectable hydrogen concentration can be improved to 1400 ppm, comparable to that achieved with electrochemical and catalytic-based hydrogen sensors. These sensor offers high spatial resolution and can be used as non-invasive detectors for various safety applications.

## 5. Conclusions

This paper analyzed the application of 3ω sensors for hydrogen concentration measurement in air mixture. To derive sensor designs sensitive to hydrogen concentrations below the lower flammability limit of 4%, we considered low thermal conductivity substrates and suspended-type 3ω sensors. Theoretical calculations were performed to compute and compare the temperature amplitudes of 3ω sensors based on hydrogen concentration, AC frequency, and microheater width. Assuming a 2% uncertainty in temperature amplitude measurement for 3ω sensors, suspended-type sensors exhibited the best performance, with at least 0.3% hydrogen concentration detection capabilities. As the thermal resistance of the substrate decreases, polyimide and SiO_2_ substrate-based 3ω sensors are predicted to achieve minimum hydrogen detection concentrations of 3% and 18%, respectively. By narrowing down the range for AC frequency fitting in thermal conductivity and implementing low-frequency noise reduction, it is anticipated that the measurement uncertainty of 3ω signals can be reduced to 1%. In such a scenario, suspended sensors are expected to achieve detection levels as low as ~1400 ppm, comparable to those achieved with electrochemical hydrogen sensors. This study highlights the potential of thermal-conductivity-based hydrogen sensors for detecting hydrogen leaks with high spatial resolution and accuracy compared to conventional techniques.

## Figures and Tables

**Table 1:** Calculation parameters and conditions used for the detection of hydrogen in air.

Parameter	Condition and Range
Substrate type	SiO_2_, polyimide substrates and suspended sensors
Microheater width	4, 10, and 40 μm
AC frequency	0.1~100 Hz
Hydrogen mole fraction in air	0~20%

## Data Availability

Not applicable.

## References

[1] Aziz M. (2021). Liquid hydrogen: A review on liquefaction, storage, transportation, and safety. Energies.

[2] Rivard E., Trudeau M., Zaghib K. (2019). Hydrogen storage for mobility: A review. Materials.

[3] Abe J.O., Popoola A.P., Ajenifuja E., Popoola O.M. (2019). Hydrogen energy, economy and storage: Review and recommendation. Int. J. Hydrogen Energy.

[4] Najjar Y.S. (2013). Hydrogen safety: The road toward green technology. Int. J. Hydrogen Energy.

[5] Buttner W.J., Post M.B., Burgess R., Rivkin C. (2011). An overview of hydrogen safety sensors and requirements. Int. J. Hydrogen Energy.

[6] Hübert T., Boon-Brett L., Black G., Banach U. (2011). Hydrogen sensors—A review. Sens. Actuators B.

[7] Ndaya C.C., Javahiraly N., Brioude A. (2019). Recent advances in palladium nanoparticles-based hydrogen sensors for leak detection. Sensors.

[8] Chauhan P.S., Bhattacharya S. (2019). Hydrogen gas sensing methods, materials, and approach to achieve parts per billion lever detection: A review. Int. J. Hydrogen Energy.

[9] Luo Y., Zhang C., Zheng B., Geng X., Debliquy M. (2017). Hydrogen sensors based on noble metal doped metal-oxide semiconductor: A review. Int. J. Hydrogen Energy.

[10] Korotcenkov G., Han S.D., Stetter J.R. (2009). Review of electrochemical hydrogen sensors. Chem. Rev..

[11] Oh D.W. (2022). Analysis on measurement of hydrogen concentration in air mixture using 3 omega method. Int. J. Nanotechnol..

[12] Berndt D., Muggli J., Heckel R., Rahiman M.F., Lindner M., Heinrich S., Plöchinger H., Schreiner R. (2022). A Robust Miniaturized Gas Sensor for H_2_ and CO_2_ Detection Based on the 3ω Method. Sensors.

[13] Berndt D., Muggli J., Wittwer F., Langer C., Heinrich S., Knittel T., Schreiner R. (2020). MEMS-based thermal conductivity sensor for hydrogen gas detection in automotive applications. Sens. Actuators A.

[14] Yusibani E., Woodfield P.L., Fujii M., Shinzato K., Zhang X., Takata Y. (2009). Application of the three-omega method to measurement of thermal conductivity and thermal diffusivity of hydrogen gas. Int. J. Thermophys..

[15] Oh D.-W., Jain A., Eaton J.K., Goodson K.E., Lee J.S. (2008). Thermal conductivity measurement and sedimentation detection of aluminum oxide nanofluids by using the 3ω method. Int. J. Heat Fluid Flow.

[16] Gauthier S., Giani A., Combette P. (2013). Gas thermal conductivity measurement using the three-omega method. Sens. Actuators A.

[17] Cahill D.G. (1990). Thermal conductivity measurement from 30 to 750 K: The 3ω method. Rev. Sci. Instrum..

[18] Oh D.-W. (2017). Thermal conductivity measurement of liquids by using a suspended microheater. Int. J. Thermophys..

[19] Lee S.M., Cahill D.G. (1997). Heat transport in thin dielectric films. Rev. Sci. Instrum..

[20] Kim H.S., Ko W.J., Oh D.-W. (2021). Analysis of thickness and interfacial thermal resistance of Au microheater on glass. High Temp.–High Pressures.

[21] Bauer M.L., Norris P.M. (2014). General bidirectional thermal characterization via the 3ω technique. Rev. Sci. Instrum..

[22] Mathur S., Tondon P.K., Saxena S.C. (1967). Thermal conductivity of binary, ternary and quaternary mixtures of rare gases. Mol. Phys..

[23] Oh D.-W. (2020). Thermal property measurement of nanofluid droplets with temperature gradients. Energies.

